# Synergetic effect of high dose rate radiations (10× FFF/2400 MU/min/10 MV x‐rays) and paclitaxel selectively eliminates melanoma cells

**DOI:** 10.1002/cnr2.1733

**Published:** 2022-10-14

**Authors:** Niraj Lodhi, Poonam Nagpal, Sreeja Sarojini, Michaela Keck, Yuk Ming Chiu, Zeenath Parvez, Laura Adrianzen, K. Stephen Suh

**Affiliations:** ^1^ The Genomics and Biomarkers Program Hackensack University Medical Center, Hackensack Meridian Health Hackensack New Jersey USA; ^2^ College of Natural, Applied, and Health Sciences Kean University Union New Jersey USA; ^3^ DiagnoCine Hackensack New Jersey USA

**Keywords:** apoptosis, combination radio‐chemotherapy, flash radiation therapy, melanoma, PARP1, synthetic lethality

## Abstract

**Background:**

Melanoma is one of the most aggressive cancers, with 1.6% of total cancer deaths in the United States. In recent years treatment options for metastatic melanoma have been improved by the FDA approval of new therapeutic agents. However, these inhibitors‐based therapies are non‐specific and have severe toxicities, including hyperkeratosis, photosensitivity, hepatitis, arthralgia, and fatigue.

**Aims:**

The aim of this study is to determine the synthetic lethal effect (paclitaxel and radiations) on melanoma cells and reduce the total radiation doses by increasing the dose rates up to 2400 MU/min.

**Methods and Results:**

We previously reported a radiation treatment (10 MV x‐rays, 10X‐FFF, dose rate 2400MU/min, low total dose 0.5 Gy) that kills melanoma cells with 80% survival of normal HEM in vitro. In this study, we extended the radiation cycle up to four and included paclitaxel treatment to study the synthetic lethal effect on melanoma and two other normal primary cells, HDF and HEK. Cells were treated with paclitaxel prior to the radiation at a dose rate of 400 and 2400 MU/min with a total radiation dose of only 0.5 Gy. Mitochondrial respiration assay, DNA damage assay, and colony formation assays were performed to study apoptosis and cell death induction. Four days of consequent radiation treatment with paclitaxel significantly reduces the survival of melanoma cells by inducing apoptosis and mitochondrial damage. After treatment, excessive DNA damage in melanoma cells leads to an increase in the expression of pro‐apoptotic genes (Caspase‐3) and a decrease in the expression of DNA repair gene (PARP1) and anti‐apoptotic gene (Bcl‐2) to activate the apoptosis pathway. The combination of paclitaxel and radiation reduces the survival of melanoma cells colonies compared to radiation alone.

**Conclusion:**

Our study indicates that radiations with paclitaxel have a potential synthetic lethal effect on melanoma cells and can be developed as a melanoma therapy without toxicities or harmful effects on normal primary skin cells.

## 1 INTRODUCTION

After identifying BRAF mutation, one of the important and major drivers of melanoma, it has led to the development of targeted therapies, including immunotherapy, single agent or combination chemotherapy and radiotherapy.^1^ However, the 10‐year survival rate for metastatic melanoma continues to be 10%, and the recent annual number of estimated newly diagnosed cases is greater than 73 000 with death numbers reaching almost 10 000.^2^ Melanoma was initially known to be a radio‐resistant tumor,^3^ but as the radiotherapeutic measures evolved, it was proved radiosensitive, and the radiation therapy option is open.^4^ Stereotactic body radiotherapy (SBRT) uses a high dose rate of Flattening Filter Free (FFF) beams for cancer therapy because of the significantly shortened beam on time. A study of patients (n = 84) with multiple lesions (lung 75, liver 10, adrenal 6, lymph nodes 5, others 4) using 6 or 10 MV FFF beams demonstrated no acute toxicity on patients with overall survival (OS) at 94%, indicating that radiotherapy can be used in melanoma treatment.^5^ For unresectable malignant melanoma in the esophageal tract demonstrated that a combination of radio‐chemo prevents supraclavicular metastasis with favorable palliative effects.^6^

Paclitaxel (microtubules disassembly protector, blocks the progression of mitosis and triggers apoptosis) was introduced in cancer therapy decades ago and has been widely used for ovarian,^7^, ^8^, ^9^, ^10^ breast,^11^, ^12^ Kaposi‐Sarcoma (AIDS‐related)^13^, ^14^, ^15^ and lung carcinomas as a single agent or in combination with other drugs.^16^, ^17^, ^18^, ^19^, ^20^ In multiple studies, Paclitaxel was investigated for its effectiveness in treating metastatic melanoma either as a single agent or in combination with small molecule inhibitors^21^ and carboplatin^22^, ^23^, ^24^ or immune‐cytokine F8‐IL2.^25^ For non‐resection metastatic melanoma, Paclitaxel is currently in a clinical trial for first‐line therapy with other chemotherapy drugs with reporting promising primary results.^26^ The recent clinical practice uses chemo agents with radiotherapy for cancers. The Paclitaxel and cisplatin combinations were used with intensity‐modulated radiation therapy (IMRT) to treat upper esophageal carcinoma with favorable results and no significant toxicities.^27^

Based on the research of combinations of chemo and radiotherapy, we designed the protocol of four consecutive days combined treatment of Paclitaxel and high dose rate radiations on melanoma cells in extend to our previous research.^28^ We treated both melanoma and normal primary skin cells human epidermal melanocytes (HEM), human epidermal keratinocytes (HEK) and human dermal fibroblasts (HDF) for four consecutive days with a combination protocol to analyze the accumulative apoptotic effects on cancer cells in vitro. Our results suggest the apoptotic effect on melanoma cells by combining the low dose Paclitaxel with the high dose rate/low total dose (radiation) method eliminated after four treatments and without toxic effect on normal primary skin cells.

## 2 MATERIALS AND METHODS

### 2.1 Cell culture

**FIGURE 1:**
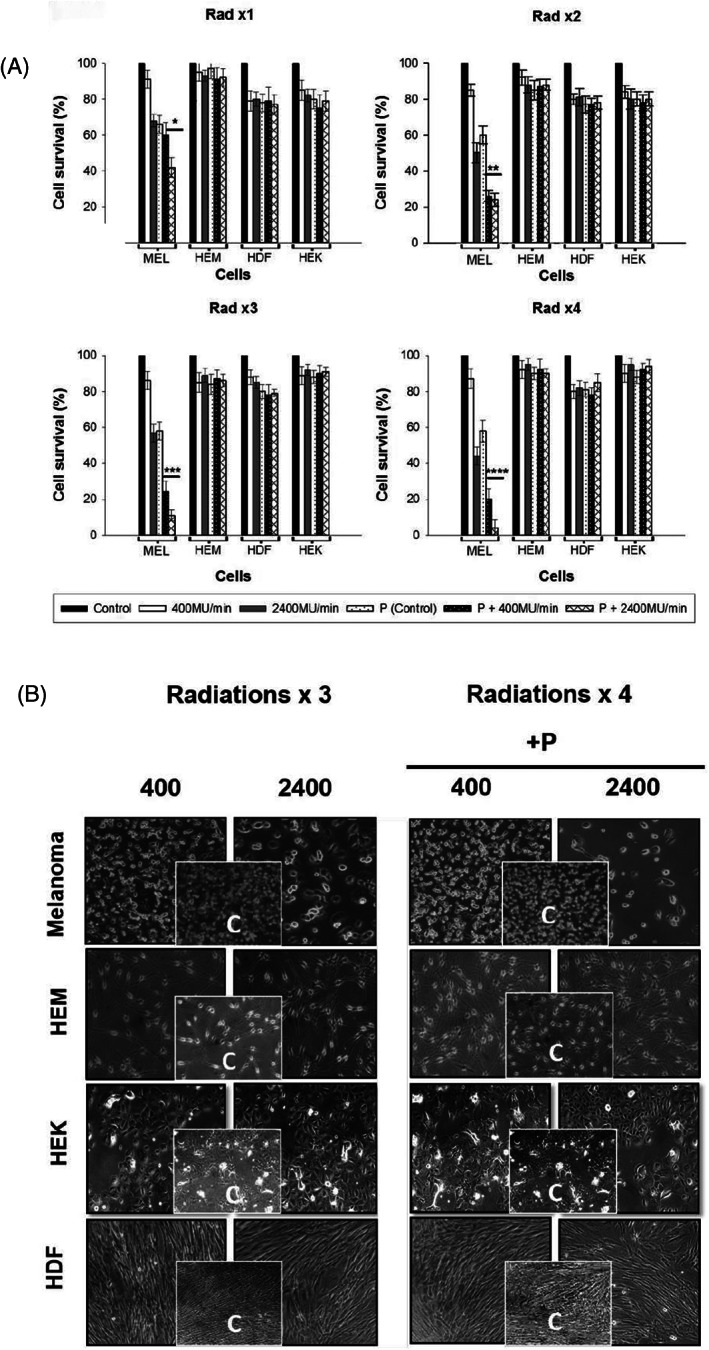
Melanoma cell line (WC00046), normal skin cells (HEM, HEK, and HDF) were irradiated (Rad ×1 to ×4) with and without Paclitaxel. (A). Melanoma cell lines showed a significant reduction in cell survival % after the third radiation in Paclitaxel treated cells. Normal cells survival was >80% even after treating with Paclitaxel. The statistical significance between the two‐dose rates for WC00046 with Paclitaxel (*) is *p* < .002, for Rad ×2 is *p* < .006 (**), Rad ×3 is *p* < .001(***), and Rad ×4 *p* < .002 (****). All experiments were repeated at least two times. (B). Live imaging of melanoma or normal cells irradiated cells. Cells in T25 culture flasks using the IDEA camera, Spot 5 by phase‐contrast microscopy after four radiations (Rad ×4). Control cells from the melanoma cell line, HEM, HEK and HDF, are given in the inset. Each cell line radiated under 400 and 2400 MU/min with and without Paclitaxel is visualized in this image. HDF, human dermal fibroblasts; HEK, human epidermal keratinocytes; HEM, human epidermal melanocytes.

Melanoma cell line WC00046 (V600E mutation in BRAF gene) (Figure 1A) purchased from Coriell Institute (Camden, NJ) was cultured in RPMI medium containing 10% FBS, 1% penicillin/streptomycin (Invitrogen, Grand Island, NY). These were originated from Wistar Institute (Philadelphia, PA), and Coriell Institute provided their authenticity, malignancy, aggressiveness and mutation data. The Coriell Institute provided their authenticity, malignancy, aggressiveness and mutation data provided their authenticity, malignancy, aggressiveness, and mutation data. HEM and culture media were purchased (ScienCell, Carlsbad, CA), and HDF and HEK were prepared as previously described.^28^, ^29^

### 2.2 Radiation treatments to cells

Cells were seeded (2× 105) in T‐25 culture flasks (BD‐Falcon), allowed to adhere overnight, and irradiated with 10 MV x‐rays at dose rates of 2400 MU/min or 400 MU/min by using TrueBeam (Varian Medical Systems, CA) with 10×‐FFF mode. The radiation dose was administered to melanoma and normal cells as shown in Figure S1 and Table S1. Each group's duplicate set of cells was pretreated with Paclitaxel (50 nM in DMSO; Sigma Aldrich) for 2 h prior to each irradiating step (Rad ×1, Rad ×2, Rad ×3, and Rad ×4). The cell culture medium was changed after 2 h of each radiation. Cells were treated with Paclitaxel only 2 h prior to each irradiation. The titrated dose of 50 nM Paclitaxel was not toxic to normal skin cells for the entire duration of the experiment. After 24 h of treatment, cells were plated. Delaying the plating of cells after irradiating allows cells a more accurate concentration versus time exposure.

### 2.3 Colony formation assays

One day after radiation, HEK, HEF, HEM, and melanoma cells were treated with trypsin, collected, and serially diluted (1:100, 1:1000, and 1:10 000) for appropriate seeding in dishes (Corning, NY) with complete media. Colonized cells (typically 21 days) were stained with hematoxylin for 30 min, fixed with 100% ethanol for 30 min, washed in water, and dried overnight for counting. Radiated cells were counted with a Beckman Coulter Counter (Brea, CA).

### 2.4 RNA isolation and quantitative PCR

The TRIzol (Invitrogen, Grand Island, NY) method was used for RNA extraction, and selected genes were amplified by qRT‐PCR with SYBR green (Qiagen, Valencia, CA) on the FAST Model 7900HT (Applied Biosystems, Carlsbad, CA). Data were analyzed using the SDS 7900HT software v2.2.2 application to determine the comparative threshold cycle (Ct) method (2^−ΔΔ*Ct*^) for calculating fold changes and standard deviations.^30^ Primer sequences are given in Table S2.

### 2.5 Cell proliferation assay

The MTS assay (CellTiter 96® AQueous One Solution, Promega, Madison, WI) was used to assess cell proliferation of radiated cells by using a Microplate reader (BioTek Synergy HT) as described by the manufacturer and based on previous publication.^28^

### 2.6 Mitochondria respiration assay

Mitotracker Red CMXRos (Invitrogen‐M7512) was used to stain cells at a final concentration of 200 nM for analyzing mitochondria activity before and after radiation. After a 15‐min incubation at 37°C and 5% CO_2_, cells were washed with PBS and fixed with 2% PFA (paraformaldehyde) for 30 min at room temperature in the dark. Fixed cells were washed with phosphate buffered saline (PBS), mounted using 4', 6‐diamidino‐2‐phenylindole (DAPI), and immediately took fluorescent images with Zeiss Fluorescent microscopy (Axiovision). Cell fluorescence was quantified by using ImageJ.

### 2.7 DNA damage assay

The EpiQuik in Situ Kit (Cat # P‐6001‐096, Epigentek, Farmingdale NY) was used to assess the phosphorylation of H2AX at Serine 139 by a colorimetric method measured at 450 nm in a 96‐well plate where the cells are grown after radiation, fixed, and permeabilized according to the manufacturer's protocol. The DNA damage assay was carried out 1 h after the Paclitaxel and the radiation treatment.

### 2.8 Western blotting

Cell lysates (Pierce IP lysis buffer cat# 87788; Invitrogen) were prepared from cell lines treated with or without Paclitaxel or radiation ×4. SDS‐PAGE gels (12%, Criterion TGX Precast Gels) were used to fractionate cell lysates and transferred onto a polyvinylidene difluoride (PVDF, Bio Rad) membrane using a Bio‐Rad transfer unit (Hercules, CA). The membranes were blocked by using 5% nonfat dry milk in TBST (10 mmol/L Tris, pH 8.0, 150 mmol/L NaCl, 0.5% Tween 20) for 60 min; they were then washed and incubated with primary antibodies against Bcl‐2 (1: 1000, #7973; Abcam, Cambridge, MA), caspase‐3 (1:2000, H‐277), PARP 1 (1:2000, #SC‐7148, SC‐ 8007; Santa Cruz Biotechnology Inc., Santa Cruz, CA) and Actin (1:10 000, AB‐6276) at 4°C overnight. Actin was used as the loading control. The membranes were then washed and incubated with a 1:5000 dilution of horseradish peroxidase‐conjugated donkey anti‐mouse IgGs secondary antibody. The blots were washed and developed using the ECL system (Pierce). Doxorubicin (Dx, topoisomerase II inhibitor) treated cell lysates were used as a positive control (+C) for Caspase‐3 and negative control for PARP1 and Bcl‐2. Samples were used from a 2400 MU/min radiations (total dose 2 Gy) Rad ×4‐treated experimental settings with (+Pac) or without (−Pac) Paclitaxel and with (T) or without (U) radiation treatment.

### 2.9 Statistical analysis

All experiments were performed a minimum of three times, and data represent the results for assays performed in triplicate or quadruplicate. Error bars represent 95% confidence intervals (CIs). All statistics were based on continuous variables by using the software STAT View, and *p* values <.05 were considered statistically significant. For comparisons between two groups, the Student's *t*‐test was applied.

## 3 RESULTS

### 3.1 Survival of melanoma cells decrease after Paclitaxel in combination with a high dose rate of 2400 MU/min (total dose 0.5 Gy) treatment

We have tested multiple melanoma cell lines and selected one cell line with a comprehensive data for malignancy, aggressiveness, and mutation data and a representative of poor clinical outcome. Viable cells were counted 4 days after four consecutive days of the treatment (Figure 1A; Rad ×1, ×2, ×3, and ×4). At the high dose rate (2400MU/min total dose 2 Gy Rad ×4) in the presence of Paclitaxel, the normal skin cells tolerated the total dose of 2 Gy (0.5 Gy ×4 = 2 Gy) for both 400MU/min and 2400MU/min at the high dose rate. In this case, the melanoma cells were selectively killed: 58% on day 1 (Rad ×1), 76% on day 2 (Rad ×2), 96% on day 3 (Rad ×3), and 98% on day 4 (Rad ×4) (Figure 1A, P+ 2400 MU/min, *p* < .005 for all 4 treatments). The cell counts after the four consecutive treatments showed that melanoma cell survival percentages decreased fivefold compared to the control (nonradiated, no Paclitaxel) for P+ 400 MU/min and 50‐fold for P+ 2400 MU/min, suggesting that 2400 MU/min in combination with Paclitaxel is highly effective for killing melanoma cells while largely preserving normal skin cells (Figure 1A). Based on bright‐field microscopy data, all normal skin cells HEM, HDF, and HEK maintained above 85% cell survival after four consecutive daily radiations with or without Paclitaxel and with 400 MU/min or 2400 MU/min dose rate (Figure 1B), suggesting that the titrated dose of Paclitaxel and 0.5 Gy of total dose delivery leads to minimal harm to normal skin cells. All skin cells retained proliferative potential and recovered fully 3 days after the fourth treatment.

### 3.2 Paclitaxel in combination with 2400 MU/min (total dose 0.5 Gy) induces greater DNA damage to melanoma cells

**FIGURE 2:**
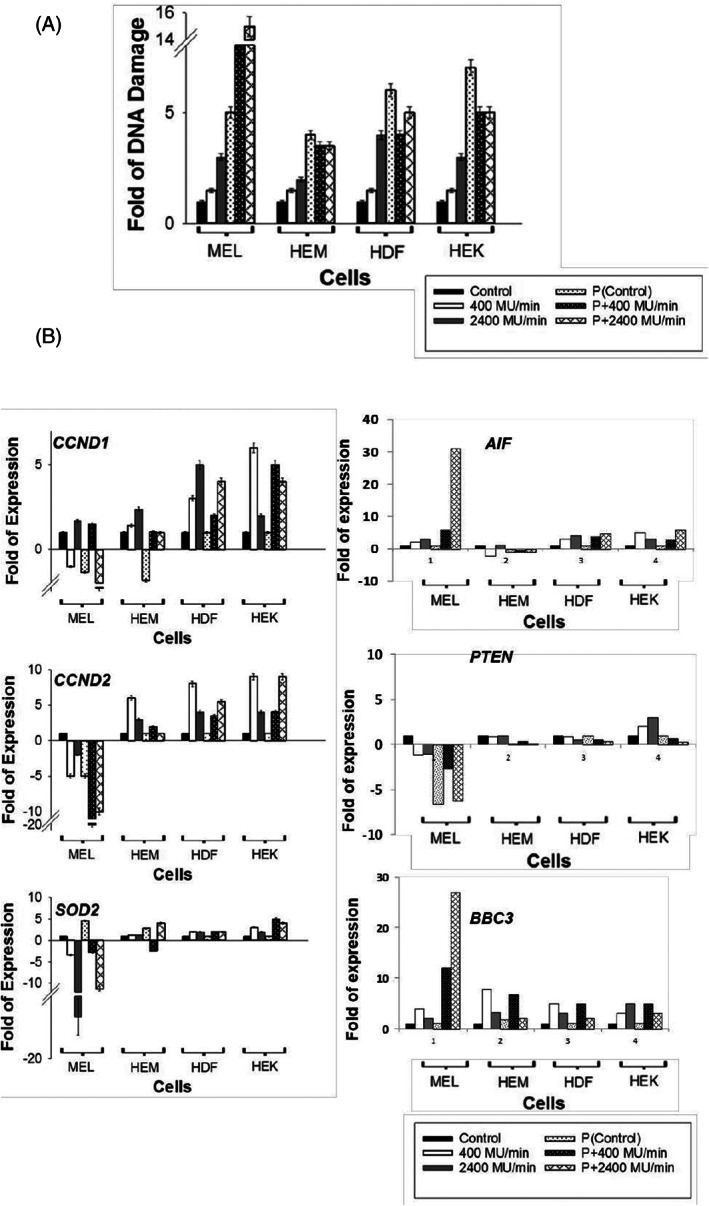
(A) DNA damage (fold) of irradiated cells with and without co‐administration of Paclitaxel for both dose rates 400 and 2400 MU/min are shown after the fourth radiation (Rad ×4). Data represent the average of four independent experiments with error bars. The statistical difference between 400 and 2400 MU/min with Paclitaxel is shown (**) *p* < .004. All experiments were performed after radiation and with or without Paclitaxel treatments and repeated at least two times. (B). mRNA expression status of cell cycle genes CCND1, CCND2, Radioprotection gene SOD2, AIF, PTEN, and BBC3 were quantified via qRT‐PCR analysis. HDF, human dermal fibroblasts; HEK, human epidermal keratinocytes; HEM, human epidermal melanocytes.

The treated cells were harvested 2 days after four consecutive treatments (Table S1) and allowed to recover for at least 24 h after each treatment prior to the subsequent radiation. Irradiating melanoma cells with 400 MU/min did not increase DNA damage, but the 2400 MU/min dose rate increased to threefold more DNA damage than the control and 400 MU/min (Figure 2A). Normal skin cells showed moderate DNA damage in cells with radiation alone, but less than Paclitaxel treated cases (Figure 2A). Residual DNA damage was lower for the combination of Paclitaxel and radiation than Paclitaxel alone (despite significant DNA damage remaining with radiation alone) in all three normal skin cells. At the same time, it is the other way in melanoma cells (Figure 2A). Despite DNA damage, cell proliferation markers CCND1 and CCND2 were moderately upregulated in treated normal skin cells but downregulated in treated melanoma cells (Figure 2B). Expression of apoptosis‐inducing factor (AIF) and pro‐apoptotic modulator BBC3 was upregulated in melanoma cells treated with 2400 MU/min dose rate with Paclitaxel. Further, the level of radioprotection gene SOD2 and tumor suppressor PTEN were downregulated in melanoma cells while upregulated the expression levels in HEM, suggesting that the DNA damage repair and radioprotection of normal skin cells are actively enhancing the survival of normal cells.

### 3.3 Paclitaxel and 2400 MU/min (total dose 0.5 Gy) radiations treatment reduce proliferation and increase mitochondrial respiration increases of melanoma cells

**FIGURE 3:**
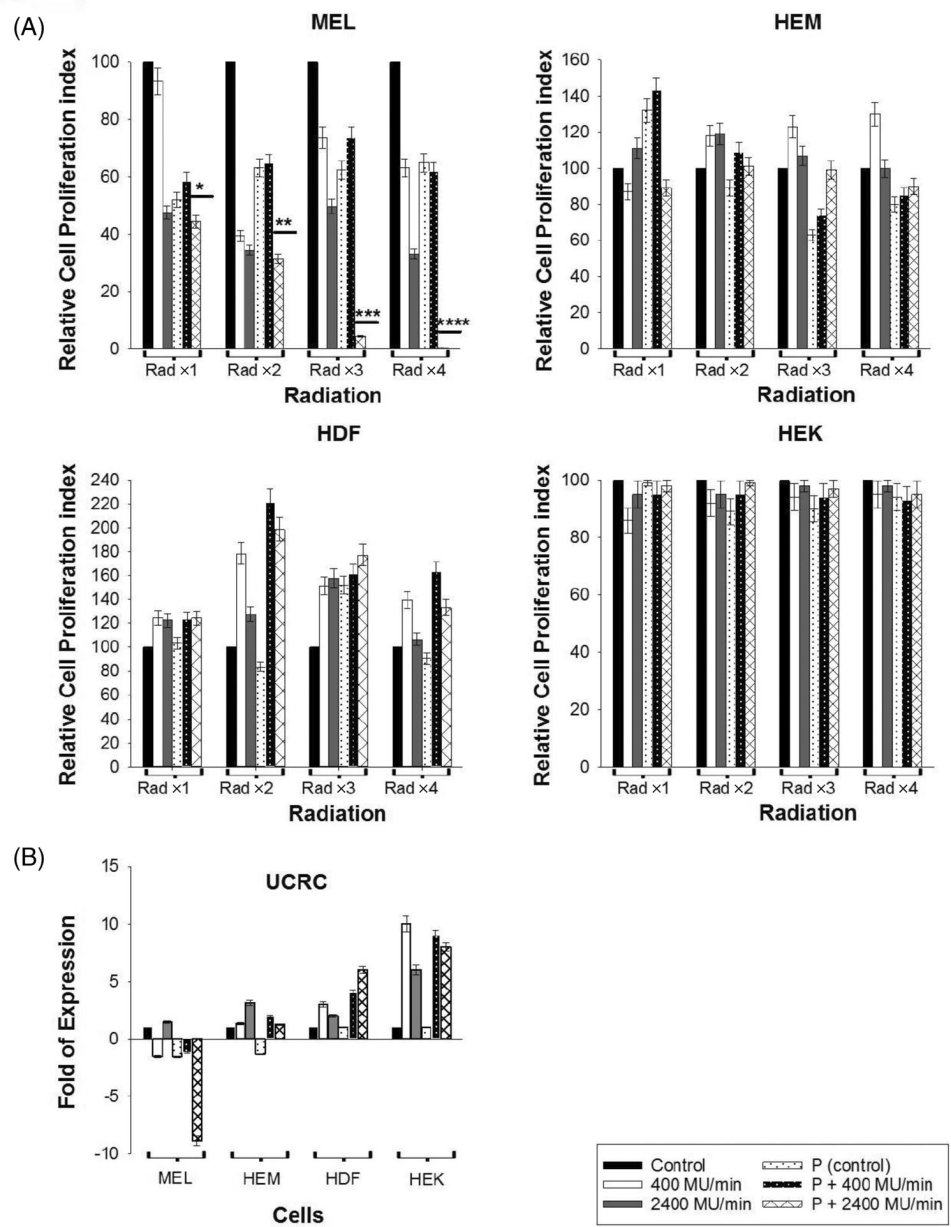
Cell proliferation was quantified by MTT assay. (A). Cell proliferation was quantified by MTT assay for irradiated cells from (A), and standard error bars and statistical *p* values are shown as (*). For the melanoma cell line, the statistical significance between 400MU/min and 2400MU/min with P (*) is *p* < .005, for Rad ×2 is *p* < .006 (**), Rad ×3 is *p* < .002 (***), and Rad ×4 *p* < .005 (****). (B) The mutational status of the mitochondrial respiration gene UCRC was analyzed using qRT‐PCR, and the average fold changes against non‐radiated control cells are shown. All experiments were repeated at least two times.

MTT assay was used to investigate cell proliferation and mitochondrial respiration of normal skin cells and melanoma cells. The cell proliferation assay using MTS reagent suggested all normal skin cells HEM, HDF, and HEK showed proliferation levels close to or above the untreated control after four radiations. In comparison, proliferation was significantly reduced in melanoma cells after four radiations. Combination treatment is effective to reduce the viability of melanoma cells – 44% (Rad ×1), 31% (Rad ×2), 4% (Rad ×3), and 0.5% (Rad ×4) versus viability of >95% after four radiations with or without Paclitaxel at a dose rate of 2400 MU/min for normal skin cells (Figure 3A).

Dose rate 2400 MU/min caused higher mitochondrial respiration than 400 MU/min, and this was directly dependent on the total dose of radiation delivered to cells. However, the respiratory chain gene (UCRC) was downregulated in melanoma cells, suggesting that the increased respiration activity after radiation was related to post‐translational activation (Figure 3B). The possible reason for lower UCRC detection of expression at the transcript level may be due to the lower number of survived cells after treatment as shown in Figure 3B.

### 3.4 Pro‐apoptotic genes are upregulated, and DNA repair PARP1 and anti‐apoptotic genes are downregulated in 2400 MU/min (total dose 2 Gy) radiated and Paclitaxel‐treated melanoma cells

**FIGURE 4:**
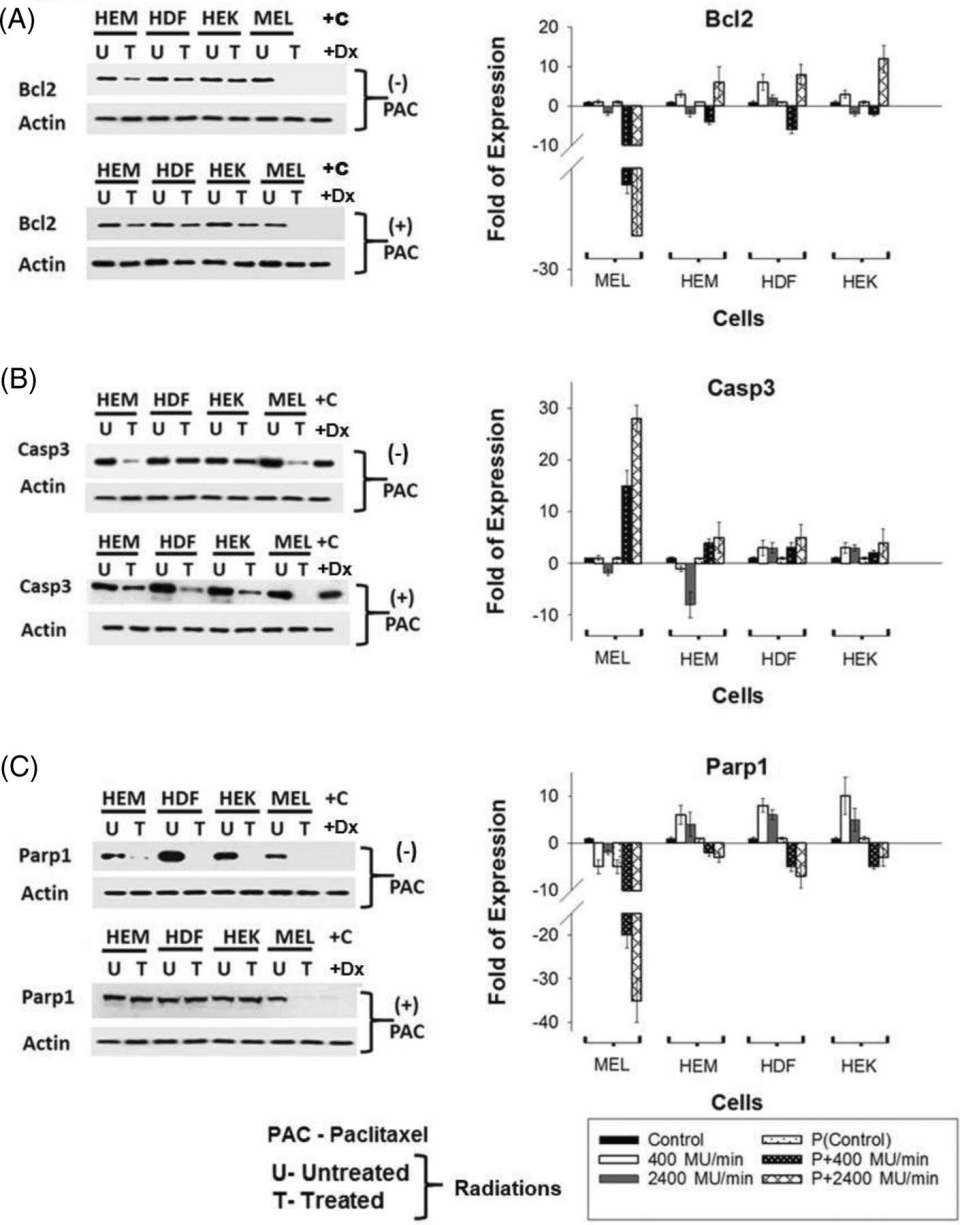
Effect of radiations and Paclitaxel on the expression of DNA repair and ant‐apoptosis genes. Western Blot analyses of Bcl‐2 (A), Caspase‐3 (B), PARP1 (C), and Doxorubicin (Dx)‐treated cell lysates were used as positive controls (+C). The samples used were from a 2400MU/min radiations (total dose 2 Gy) Rad ×4 experimental setting with (+PAC) or without (−PAC) Paclitaxel and with (T) or without (U) radiation treatment. Subsequently, qRT‐PCR analyses of the same Bcl‐2, PARP1, and Caspase‐3 were also carried out, and fold expressions were shown in graphs.

Anti‐apoptotic Bcl‐2 was marginally downregulated at protein level for primary normal melanocytes, dermal fibroblasts and keratinocytes in irradiated and Paclitaxel‐treated cases (Figure 4A). Compared to normal primary skin cells, melanoma cells showed significant downregulation on both RNA and protein levels. Pro‐apoptotic Caspase‐3 is upregulated in irradiated and Paclitaxel‐treated melanoma cells at the RNA level but showed significant downregulation on the protein level on all primary and cancerous melanoma cells (Figure 4B). While the normal skin cells retain the expression of Caspase‐3 in irradiated cells, the melanoma cells treated with Paclitaxel showed minimal expression of Caspase‐3 (Figure 4 B). The protein level of Caspase‐3 does not correlate with the RNA expression of this gene in both melanoma and control cells. The trend for the change of the protein level of Caspase‐3in response to radiation± Paclitaxel (decreased) is similar to that one for anti‐apoptotic Bcl‐2. The proapoptotic factor Caspase‐3 protein level was significantly downregulated in primary normal melanocytes, fibroblasts, and keratinocytes after radiation treatment (denoted T) and this downregulation was independent of Paclitaxel treatment, suggesting that the cell death is partially through the caspase pathway. The Caspase‐3 protein expression was minimal in melanoma cells treated with Paclitaxel.

DNA damage repair genes PARP1 was downregulated substantially in Paclitaxel‐treated and ‐irradiated melanoma cells (Figure 4C). In normal primary skin cells, the full size PARP1 was significantly decreased (increased cleaved PARP1 not shown but confirmed and correlated with downregulation of whole PARP1 in previous publication) in irradiated cells without the Paclitaxel treatment. The full‐size PARP1 protein amount was slightly decreased in normal skin cells when radiation and Paclitaxel treatments were combined, whereas Doxorubicin‐treated control cells showed absence of full length PARP1expression. Cleavage of PARP1 by caspases is considered to be a hallmark of apoptosis. Reduced expression of PARP1 in HDF and HEK in radiation‐treated groups show that apoptotic pathway is activated in these cells as well. We have reported in our previous study that PARP1 was cleaved in radiated cells (28) that suggest excessive DNA damage leads to the death of cells. Taken together, this suggests that caspases mediated apoptosis may not be the primary mechanism of cell death after this treatment, and some other mechanisms of cell death may play a major role in the killing of melanoma cells in the experiment.

**FIGURE 5:**
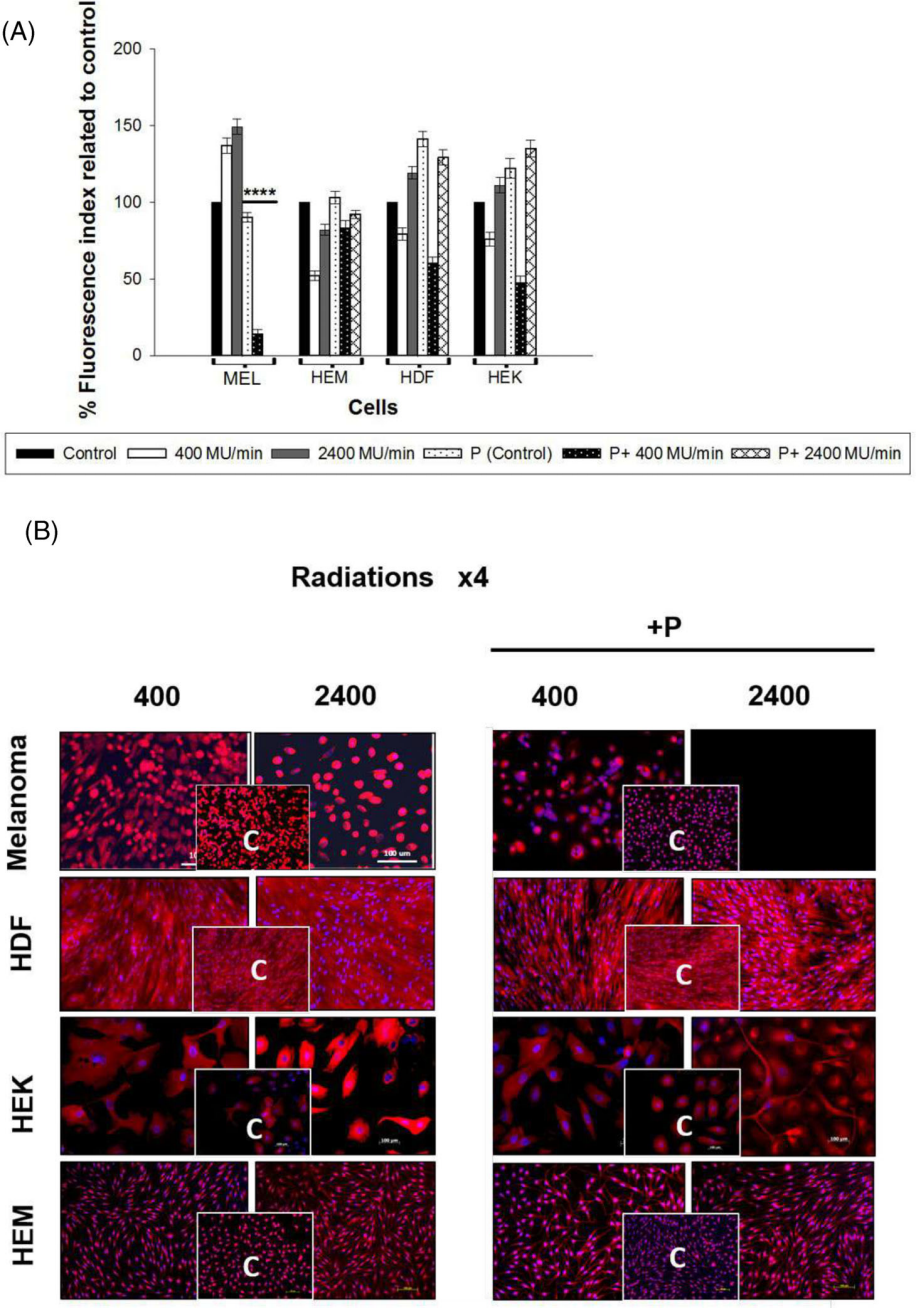
Effect of radiations and Paclitaxel on mitochondrial respiration using Mitotracker red‐fluorescent dye. (A) The average fluorescent intensity from five random fields for each experimental setting was used to calculate the relative fluorescent intensity using Image‐J software and was normalized against the average intensity of the non‐radiated control (solid black) for each cell type. The fold changes for dose rates 400 MU/min (solid white) and 2400 MU/min (solid gray) are shown with standard error bars. Paclitaxel control (P) data are represented with black dots over white; 400 MU/min + P are shown in white over black, and 2400 MU/min is in horizontal diamond bars. All experiments were repeated at least two times. The statistical significance between the two‐dose rates for WC00046 with Paclitaxel (*) is *p* < .005). (B) The melanoma cell line, HEM, HDF, and HEK cells were radiated with and without Paclitaxel four times (Rad ×1, Rad ×2, Rad ×3, and Rad ×4). The image represents Rad ×4 data. Cells were collected and seeded, and stained the following day using Mitotracker red‐fluorescent dye to detect mitochondrial respiration, and fluorescence microscopy was used for imaging at 20× magnification with scale bars. Nonradiated control cells (inset) are shown for individual radiation settings for each cell type.

A Mitotracker assay of melanoma cells treated with Paclitaxel and irradiated at 2400 MU/min (Rad ×4) showed no fluorescence, indicating cells undergoing apoptosis (Figure 5A,B). Minimal fluorescence was noticed in cells irradiated at 400 MU/min with Paclitaxel even after four radiations, suggesting that Paclitaxel induced more mitochondrial damage when co‐administered with a dose rate of 2400 MU/min in cancer cells. Primary normal cells HEM, HDF, and HEK, showed no significant difference in fluorescence between 400 and 2400 MU/min.

### 3.5 Colony formation of melanoma cells diminished after four cycles of radiations at dose rate 2400 MU/min (total dose 2 Gy) and Paclitaxel treatment

**FIGURE 6:**
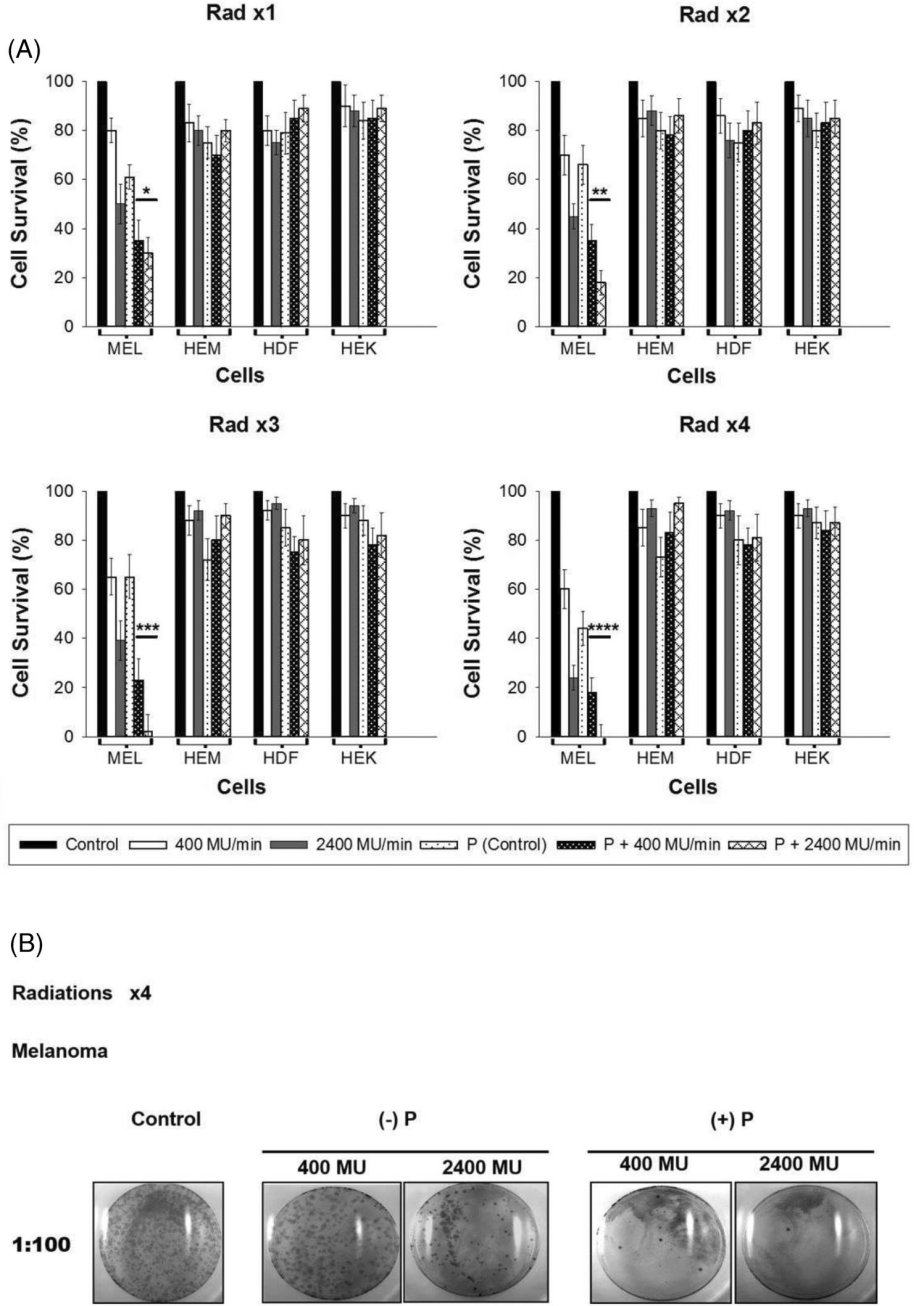
Colony formation assays after each radiation and Paclitaxel experiment. (A). Cells were counted, serially diluted, and plated on culture grade petri dishes. After 2–3 weeks colonies were stained and counted, and the survival percent (%) was calculated. The statistical significance between the two dose rates for WC00046 with Paclitaxel (*) is *p* < .001, for Rad ×2 is *p* < .004 (**), Rad ×3 is *p* < .005 (***), and Rad ×4 *p* < .002 (****). All experiments were repeated at least two times. (B). Images of colonies from the colony assay plates after staining with hemotoxylin. Melanoma cell lines were radiated with and without Paclitaxel. The image represents Rad ×4 data.

Colony formation assay has been used as the classic protocol in radiation research for determining the ability of a single cell to multiply into a colony.^32^ Assay determines the survival and proliferative potential of irradiated cells. Results show lower cell survival in 2400 MU‐treated melanoma cells than 400 MU/min (Figure 6A). Co‐administration of Paclitaxel reduced the survival of colonies to 30% (Rad ×1), 18% (Rad ×2), 2% (Rad ×3), and 0% (Rad ×4) when compared to 2400 MU/min alone (Figure 6A,B). The data showed no significant difference between the survival of colonies of irradiated and Paclitaxel‐treated cells for normal skin cells compared to radiation alone.

## 4 DISCUSSION

Melanoma is radioresistant, and radiotherapy is currently used for palliative but not curative care. In recent years, many scientific efforts have been made to compromise the DNA repair efficiency of melanoma cells, including radiation dose. The current study demonstrates the effectiveness of combining dose rate 2400 MU/min (at a low total dose of 0.5 Gy) with Paclitaxel, a cost‐effective chemotherapeutic drug. Food and Drug Administration (FDA) approved Paclitaxel induces DNA damage and mitochondrial disruptions along with microtubule inhibition.^33^ We show that melanoma cell survival is significantly reduced after four cycles of Paclitaxel and radiation treatment at the dose rate of 2400 MU/min. While minimal effect in normal skin cells HEM, HDF, and HEK (Figure 1). We measured DNA damage caused by radiations by phosphorylation of H2AXSer139 used by other research groups^34^, ^35^, ^36^, ^37^, ^38^, ^39^, ^40^, ^41^ in primary or melanoma cells. This assay shows that radiations at 2400 MU/min with Paclitaxel induce prolonged DNA damage in melanoma cells and cause sixfold more DNA damage than 2400 MU/min alone (Figure 2A). DNA damage assay (48 h after consequent irradiation) shows the DNA damage linked to apoptotic DNA cleavage (execution of apoptosis) and not the repairable DNA damage.

Excitation of mitochondria after exposure to ionizing radiation is related to the upregulation of mitochondrial electron transport chain function, which helps the cancer cells to become resistant to radiation.^42^ Paclitaxel can damage the mitochondria by altering mitochondrial respiration and thus induce mitochondrial apoptosis.^43^ We used MitoTracker probes, which passively diffuse across the plasma membrane and accumulate in active mitochondria in viable cells only.^44^, ^45^, ^46^ Further, to confirm induction of DNA Damage Response (DDR) and pro or anti‐apoptotic proteins to radiation exposure, we performed western as in Figure 4. This combination upregulates AIF, BBC3, PARP1, and Caspase‐3 genes in cancer cells to >25‐fold. Cell cycle genes (CCND1/CCND2, Figure 2), DNA damage repair genes (PARP1, Figure 4), radioprotection genes (SOD2, Figure 2), and anti‐apoptotic genes (Bcl‐2, Figure 4) were upregulated in normal skin cells. In contrast, mitochondrial and extrinsic apoptotic pathway genes (AIF and BBC3) were upregulated in Paclitaxel‐treated, irradiated cells.

The colony formation assay assesses the cell reproductive death after radiation determined by the proportion of surviving colonies for each dose administered.^47^ Our data show the decrease in cell survival for melanoma cells after Paclitaxel treatment. Results were confirmed with the MTS assay, where a decrease in viability of melanoma cells was recorded when combined with Paclitaxel, while normal cells retained their viability >95% (Figure 3A).

When melanoma cells are concurrently treated with Paclitaxel, cell number, and mitochondrial efficiency decrease, and cells show low fluorescence for 400 MU/min and no fluorescence for 2400 MU/min after four radiations. The data suggest that Paclitaxel induces greater mitochondrial damage when co‐administered with a dose rate of 2400 MU/min in melanoma cells. As shown in Figure 5A,B, the viability of Paclitaxel and radiation exposure cells is very low or none. The cancer cells with lowered DNA repair capacity, especially the aggressively dividing melanoma cells, exhibit higher lethal effects with 2.4 Gy/min than 0.4 Gy/min radiation, suggesting the higher grade caused heavier damage to the DNA, and the synergy of lethal effect is greater when radiation was combined with chemo agent such as Paclitaxel.

Coadministration of Paclitaxel completely inhibited the survival of colonies in melanoma cells after four radiations at a dose rate of 2400 MU/min (Figure 6). Similar to the standard radiotherapy (400 MU/min), melanoma cells exhibit resistance to 400 MU/min in Rad ×1 to Rad ×4 (total dose 2 Gy), even in the presence of Paclitaxel. However, our studies suggest that high dose rate radiation (2400 MU/min of total dose 2 Gy) completely abolishes the melanoma cells in combination with Paclitaxel. In other studies, Paclitaxel was used with chemo and radiotherapy.^31^, ^48^ These studies are in the clinical trials phase and their primary outcome measures are to improve the overall response rate (ORR), proliferation free state (PFS), or overall survival (OS). Paclitaxel‐based chemo‐radiotherapy for non‐small cell lung cancer (NSCLC) patients demonstrated that minimum toxicity and an optimal radio–‐chemo combination regimen are yet to be established with further studies.^31^ Paclitaxel was also used in melanoma frontline therapy as a single agent or in combination with carboplatin (DNA‐damaging agent) immunotherapeutic drugs (Ipilimumab) in phase II clinical trial to find an innovative approach to increase survival rates of patients.^48^

Our study is a new approach for radiotherapy by increasing the dose rate and reducing the total dose (0.5 Gy) to lower the toxic effects of radiations in a combination of Paclitaxel to treat BRAF mutant patients. In general, patients get the 40–60 Gy of radiation dose in 4–5 cycles, and that causes the severe side effects of other skin problems, fatigue and radiation pneumonitis. Although this is in vitro study, the death of melanoma cells is significant, and the data showed no toxic effects on primary cells at a total dose of 2 Gy in four cycles.

In conclusion, our study is a significant finding that supports the use of radiotherapy with chemo for melanoma, where standard radiotherapy fails to kill melanoma cells due to radio resistance. Important therapeutic advantage is that normal keratinocytes, dermal fibroblasts, and melanocytes were not largely harmed after the fourth cycle of dose rate of 400 or 2400 MU/min radiations with or without Paclitaxel. Although future studies using in vivo models will be required to prove this concept for clinical settings, this study indicates the effectiveness of Paclitaxel when combined with a high dose rate of radiation (2400 MU/min) and a low total dose (0.5 Gy) in melanoma therapy.

## AUTHOR CONTRIBUTIONS

**Poonam Nagpal:** Formal analysis (supporting); methodology (supporting); validation (supporting); visualization (supporting); writing – review and editing (supporting). **Sreeja Sarojini:** Formal analysis (equal); investigation (equal); methodology (equal); project administration (equal); resources (equal); supervision (equal); validation (equal); visualization (equal); writing – original draft (equal). **Michaela Keck:** Formal analysis (supporting); methodology (supporting); resources (supporting); validation (supporting); visualization (supporting); writing – original draft (supporting). **Yuk Ming Chiu:** Formal analysis (supporting); methodology (supporting); validation (supporting); visualization (supporting); writing – original draft (supporting). **Zeenath Parvez:** Formal analysis (supporting); methodology (supporting); resources (supporting); validation (supporting); visualization (supporting); writing – original draft (supporting). **Laura Adrianzen:** Formal analysis (supporting); methodology (supporting); resources (supporting); validation (supporting); visualization (supporting); writing – original draft (supporting).

## CONFLICT OF INTEREST

The authors disclosed no potential conflicts of interest.

## Supporting information


**Supplementary Figure S1.** Radiation dose administration to melanoma or normal cells.
Cell groups were divided into one radiation (Rad ×1, on day 1), second radiation (Rad ×2; day 2), third radiation (Rad ×3; day 3), and fourth radiation (Rad ×4; day 4).

**Supplementary Table S1.** Cell groups for Paclitaxel or Paclitaxel with irradiation treatment.

**Supplementary Table S2.** Primer sequences used in this study.

## Data Availability

Data sharing is not applicable to this article as no new data were created or analyzed in this study.
