# Supplementary figures and images for: Synergetic effect of high dose rate radiations (10× FFF/2400 MU/min/10 MV x‐rays) and paclitaxel selectively eliminates melanoma cells
Source: Cancer Rep (Hoboken). 2022 Oct 14;6(2):e1733. doi: 10.1002/cnr2.1733 (PMC9940010; doi:10.1002/cnr2.1733)

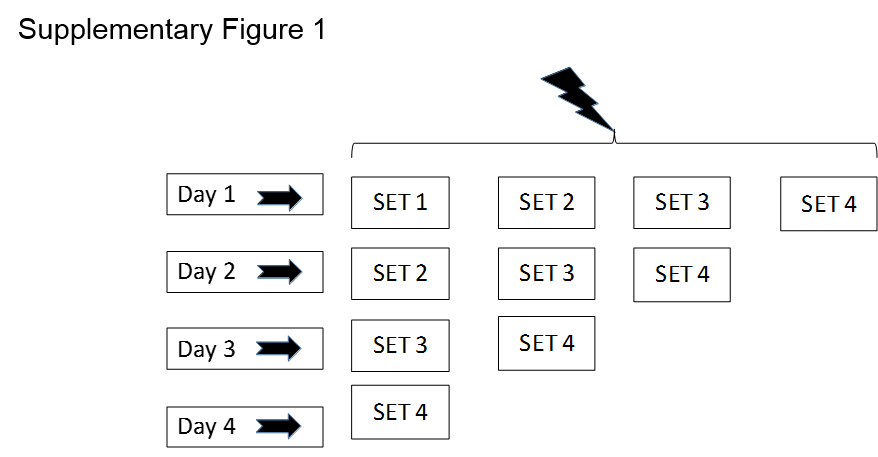

Supplement: Supplementary file 1 — Supplementary Figure S1. Radiation dose administration to melanoma or normal cells. Cell groups were divided into one radiation (Rad ×1, on day 1), second radiation (Rad ×2; day 2), third radiation (Rad ×3; day 3), and fourth radiation (Rad ×4; day 4). [file CNR2-6-e1733-s002.docx]
