# Supplementary material for: Synergetic effect of high dose rate radiations (10× FFF/2400 MU/min/10 MV x‐rays) and paclitaxel selectively eliminates melanoma cells
Source: Cancer Rep (Hoboken). 2022 Oct 14;6(2):e1733. doi: 10.1002/cnr2.1733 (PMC9940010; doi:10.1002/cnr2.1733)
Supplement: Supplementary file 2 — Supplementary Table S1. Cell groups for Paclitaxel or Paclitaxel with irradiation treatment. [file CNR2-6-e1733-s001.docx]

**Table 1. Cell groups for Paclitaxel or Paclitaxel with irradiation treatment**

|  | Control | Paclitaxel (50nM) | 400MU/0.5Gy | 400MU/0.5Gy  + Paclitaxel (50nM) | 2400MU/0.5Gy | 2400MU/0.5Gy  + Paclitaxel (50nM) |
| --- | --- | --- | --- | --- | --- | --- |
| Melanoma cells | 1*4 | 1*4 | 1*4 | 1*4 | 1*4 | 1*4 |
| HEM | 1*4 | 1*4 | 1*4 | 1*4 | 1*4 | 1*4 |
| HDF | 1*4 | 1*4 | 1*4 | 1*4 | 1*4 | 1*4 |
| HEK | 1*4 | 1*4 | 1*4 | 1*4 | 1*4 | 1*4 |
