# Supplementary material for: Synergetic effect of high dose rate radiations (10× FFF/2400 MU/min/10 MV x‐rays) and paclitaxel selectively eliminates melanoma cells
Source: Cancer Rep (Hoboken). 2022 Oct 14;6(2):e1733. doi: 10.1002/cnr2.1733 (PMC9940010; doi:10.1002/cnr2.1733)
Supplement: Supplementary file 3 — Supplementary Table S2. Primer sequences used in this study. [file CNR2-6-e1733-s003.docx]

| **Primer sequences used in this study** | |
| --- | --- |
| Casp3 f | gaactggactgtggcattga |
| Casp3 r | tcaagcttgtcggcatactg |
| Bcl-2 f | ttccagagacatcagcatgg |
| Bcl-2 r | tgtccctaccaaccagaagg |
| PARP 1 f | gctcctgaacaatgcagaca |
| PARP 1 r | tcctgatgatctcggcttct |
| SOD2 f | gggagatgttacagcccagata |
| SOD2 r | agtcacgtttgatggcttcc |
| UCRC f | attcgctgttggcaagaaac |
| UCRC r | tttgcagagggctttgaagt |
| PTEN f | gaatggagggaatgctcaga |
| PTEN r | cgcaaacaacaagcagtgac |
| CCND1 f | ctctcattcgggatgattgg |
| CCND1 r | gtgagctggcttcattgaga |
| GAPDH f | tcaccagggctgcttttaac |
| GAPDH r | atgacaagcttcccgttctc |
| CCND2 f | tgcagaaggacatccaaccc |
| CCND2 r | gccaagaaacggtccaggta |

**Supplementary Table 2**
